# The COVID-19 Tracheostomy Experience at a Large Academic Medical Center in New York during the First Year

**DOI:** 10.3390/jcm13072130

**Published:** 2024-04-07

**Authors:** Dhruv Patel, Anthony Devivo, Evan Leibner, Atinuke Shittu, Usha Govindarajulu, Pranai Tandon, David Lee, Randall Owen, Gustavo Fernandez-Ranvier, Robert Hiensch, Michael Marin, Roopa Kohli-Seth, Adel Bassily-Marcus

**Affiliations:** 1 Institute for Critical Care Medicine, Icahn School of Medicine at Mount Sinai, New York, NY 10029, USA; dhruv.patel@mountsinai.org (D.P.); anthony.devivo@mountsinai.org (A.D.); adel.bassily-marcus@mountsinai.org (A.B.-M.); 2 Department of Surgery, Icahn School of Medicine at Mount Sinai, New York, NY 10029, USA; 3 Department of Medicine, Icahn School of Medicine at Mount Sinai, New York, NY 10029, USA; 4 Department of Emergency Medicine, Icahn School of Medicine at Mount Sinai, New York, NY 10029, USA; 5 Center for Biostatistics, Department of Population Health Science and Policy, Icahn School of Medicine at Mount Sinai, New York, NY 10029, USA; 6 Department of Medicine, Division of Pulmonary, Critical Care, and Sleep Medicine, Icahn School of Medicine at Mount Sinai, New York, NY 10029, USA

**Keywords:** COVID-19, tracheostomy, ICU length of stay, ARDS

## Abstract

**Background:** New York City was the epicenter of the initial surge of the COVID-19 pandemic in the United States. Tracheostomy is a critical procedure in the care of patients with COVID-19. We hypothesized that early tracheostomy would decrease the length of time on sedation, time on mechanical ventilation, intensive care unit length of stay, and mortality. **Methods:** A retrospective analysis of outcomes for all patients with COVID-19 who underwent tracheostomy during the first year of the COVID-19 pandemic at the Mount Sinai Hospital in New York City, New York. All adult intensive care units at the Mount Sinai Hospital, New York. Patients/subjects: 888 patients admitted to intensive care with COVID-19. Results: All patients admitted to the intensive care unit with COVID-19 (888) from 1 March 2020 to 1 March 2021 were analyzed and separated further into those intubated (544) and those requiring tracheostomy (177). Of those receiving tracheostomy, outcomes were analyzed for early (≤12 days) or late (>12 days) tracheostomy. Demographics, medical history, laboratory values, type of oxygen and ventilatory support, and clinical outcomes were recorded and analyzed. **Conclusions:** Early tracheostomy resulted in reduced duration of mechanical ventilation, reduced hospital length of stay, and reduced intensive care unit length of stay in patients admitted to the intensive care unit with COVID-19. There was no effect on overall mortality.

## 1. Introduction

The initiation and use of a tracheostomy in the intensive care unit (ICU) has become an increasingly common procedure in patients who require prolonged critical care, have difficulty weaning from the ventilator, and/or have failed trials of extubation. Over one-third of patients who require greater than two days of mechanical ventilation in the ICU require tracheostomy [1]. Over the past several decades, extensive literature has been published evaluating the potential advantages of tracheostomy over prolonged endotracheal intubation, as well as the timing for when this procedure should be performed. Tracheostomy has repeatedly been shown to significantly decrease ventilator-associated pneumonia and the duration of time requiring mechanical ventilation, shorten duration of continuous sedation, and decrease the length of stay in the ICU when performed within the first seven to ten days of endotracheal intubation [2,3,4,5]. While it is a continued point of contention within the field of critical care, there is some evidence supporting an overall improvement in long-term mortality in patients who undergo early tracheostomy [5,6].

In light of the worldwide increase in patients developing acute respiratory distress syndrome (ARDS) after contracting the SARS-CoV-2 virus leading to the development of COVID-19 pneumonia, the critical care community has seen a subsequent increase in patients requiring prolonged mechanical ventilation, high-dose sedation, and lengthened ICU stays, often inevitably requiring tracheostomy. Previous data suggest that up to 15% of hospitalized patients with COVID-19 pneumonia require intubation and mechanical ventilatory support [7]. Furthermore, data from a large institution in the southern United States from 2020 found that over 80% of patients who met the criteria for ICU admission required mechanical ventilatory support [8]. While the majority of patients admitted to ICUs in the setting of COVID-19 pneumonia, particularly during the initial outbreak of the SARS-CoV-2 pandemic, required mechanical ventilation, upwards of 60% needed long-term mechanical ventilatory support necessitating tracheostomy [9]. The vast number of patients requiring tracheostomy and long-term mechanical ventilatory support has raised numerous questions regarding procedure safety, appropriate timing, and long-term outcome benefits in COVID-19.

While there is substantial evidence supporting the advantages and benefits of early tracheostomy in the setting of acute hypoxic respiratory failure, COVID-19 presented a plethora of unique circumstances that caused initial delays in the performance of tracheostomies. Early on in the COVID-19 pandemic, delays in tracheostomy occurred in the setting of safety concerns for the team performing the procedure, which is considered aerosol-generating [10]. Given these concerns, initial guidelines suggested delaying tracheostomy until at least day ten and then only if the patient was showing signs of clinical improvement [11]. These concerns have since been mitigated with evidence reassuring that tracheostomy can be performed safely and with low rates of COVID-19 transmission when appropriate enhanced personal protective equipment (PPE) is utilized [7,12,13,14]. A retrospective study from the United Kingdom assessed viral transmission in their tracheostomy team after the performance of one hundred tracheostomies over the course of six weeks and found no healthcare provider transmission attributable to the procedure [9]. A meta-analysis of current literature assessing outcomes of tracheostomies in the setting of COVID-19 pneumonia found that 94.8% of studies reported no SARS-CoV-2 transmission amongst healthcare provider teams who performed the tracheostomies [7].

Aside from safety concerns related to potential viral transmission, the performance of early tracheostomies for patients with COVID-19 pneumonia has been hindered by the prolonged critical acuity, high ventilator requirements, and profoundly high mortality initially found to be associated with COVID-19 patients requiring mechanical ventilatory support [15,16]. Prior to the COVID-19 pandemic, high ventilator demand was often defined as a Positive End Expiratory Pressure of greater than or equal to 10–12 cm H20 or an FIO2 > 50–70% [17,18]. As subsequent data have become available throughout the pandemic, it has been found that tracheostomies can be performed safely on patients who are critically ill, with reasonably high ventilator requirements, and without increased complications related to oxygenation [19]. In addition, despite initial mortality reports of patients with ARDS in the setting of COVID-19 pneumonia being nearly 80%, current data suggest that improved treatment modalities, resources, and overall management have decreased ICU mortality despite mechanical ventilator requirements to between 26.5 and 44% [8,9,20].

While the international academic community has accrued a substantial amount of data on the benefits of early tracheostomy in regard to ICU length of stay, as well as ventilator-free days, questions remain regarding the optimal time to perform an early tracheostomy in order to obtain the best possible outcomes. This is particularly important when taking into consideration the immense quantities of sedation and long-term paralysis many COVID-19 patients have been noted to require in order to maintain adequate gas exchange in the setting of ventilator dyssynchrony [21,22]. Prior to the COVID-19 pandemic, evidence with regard to the timing of tracheostomy in patients who require prolonged mechanical ventilation suggested a significant decrease in continuous intravenous sedation when performed early [23,24,25]. Given the profoundly high dosages of continuous intravenous sedatives that have been noted to be required to achieve adequate sedation and ventilator synchrony in the setting ARDS from COVID-19 pneumonia, ascertaining adequate data to answer the question of whether not early tracheostomy can assist in decreasing sedative requirements particularly prudent [22,26]. In the retrospective data we have collected and analyzed below, we present some evidence to support the notion that early tracheostomy, when compared to delayed, can facilitate decreasing days of continuous intravenous sedation, as well as ICU length of stay.

## 2. Methods

We searched our medical records for all patients admitted with a positive SARS-CoV-2 PCR test between 1 March 2020 and 1 March 2021. For all relevant baseline covariates, we computed means, standard deviation, and range (min and max) for all continuous ones and frequency and percent for categorical ones. In addition, we compared each covariate between groups, which were defined by dichotomizing tracheostomy at day 12, by either using a two-sided two-sample *t*-test for a continuous covariate or a Pearson chi-square test of association for a categorical covariate. We then compared each of the following outcomes between groups: continuous sedation, hospital LOS or ICU LOS, days on ventilator from initial intubation to date weaned, and days of continuous paralytics. We also created a histogram of tracheostomies by day. Finally, we created a Kaplan–Meier survival probability plot for the time to death with right censoring. All analyses were conducted at the 0.05 level of significance and were analyzed in SAS Version 9.4 and also R version 4.0.3. The study was reviewed by the Mount Sinai Hospital IRB # 20-00584.

## 3. Results

During the first year of the COVID-19 pandemic in New York City, between 1 March 2020 and 1 March 2021, the Mount Sinai Hospital admitted 3672 patients with COVID-19 pneumonia, of which 888 (24.8%) patients required an ICU stay, among which 544 (14.81%) required mechanical ventilation. Among the 544 patients who required mechanical ventilation, 177 (32.53%) underwent tracheostomy. The demographics of these patients are shown in Table 1. Among the patients who were on mechanical ventilation, a total of 177 (32.53%) underwent tracheostomy. Most of them were bedside percutaneous tracheostomy and were performed by Intensivists, General Surgery, Cardiothoracic Surgery, and Otolaryngology. Table 2 shows the demographics of the patients in each group along with the degree of elevation in inflammatory markers as well the extent of impact the disease process had on gas exchange, including the highest PCO2 as well as lowest PaO2 and PaO2:FiO2 ratios. Of the total tracheostomies performed in the setting of severe COVID-19 pneumonia during this timeframe, 74 (41.81%) were carried out on or before day 12 of mechanical ventilation, while 103 (58.19%) were carried out after day 12; further details can be seen in Table 3.

We noted that patients who had tracheostomy performed on or before 12 days of mechanical ventilation spent significantly less time on sedation (10.21 vs. 15.64, 95% CI of difference: −6.85, –4.00) before tracheostomy. Post-tracheostomy patients with early tracheostomy spent 9.64 days on sedation compared to 8.13 days for patients who underwent tracheostomy after 12 days (95% CI of difference: −1.70, 4.72). However, when looking at total days requiring continuous sedation while on mechanical ventilator, the early tracheostomy group spent significantly fewer days (19.89 days, 95% CI: 16.72, 23.00) compared to the later tracheostomy group (23.81 days, 95% CI: 21.71, 25.92). Similarly, the early tracheostomy group required paralytics for a mean of 5.79 days as compared to the late tracheostomy group, which required paralytics for 9.66 days (95% CI: −6.11, −1.64). This difference was also reflected in the Intensive care unit length of stay (LOS) which was 23.66 days (95% CI: 14.31, 33.01) in the early tracheostomy group compared to 67.91 days (95% CI: 0, 139.8) in the late tracheostomy group; and also in the hospital length of stay (LOS) which was 33.2 days (95% CI: 19.25, 47.16) in the early tracheostomy group, compared to 88.8 days (95% CI: 18.48, 159.1) in the late tracheostomy group. A total of 25 patients in the early tracheostomy group and 43 patients in the late ventilator group were off the mechanical ventilator at the end of the follow-up period. The early tracheostomy group spent an average of 22.9 days (95% CI: 18.65, 27.27) on the mechanical ventilator, which was significantly shorter than the late tracheostomy group, which was 34.72 days (95% CI: 30.43, 39.00). There were four deaths in the early tracheostomy group and seven in the late tracheostomy group. After adjusting for people who died, patients in the early group spent 23.04 days (95% CI: 17.95, 28.14) vs. 34.44 days (95% CI: 29.84, 39.04) in the late tracheostomy group from intubation to being weaned off mechanical ventilation, as can be seen in Table 3. Additionally, the dispositions of the patients based on early vs. late tracheostomy can be seen in Table 4.

## 4. Discussion

There remains a lack of evidence to guide whether patients with ARDS in the setting of COVID-19 pneumonia would incur the same benefits that have previously been found to be associated with early tracheostomy in patients requiring prolonged mechanical ventilation. As the pandemic has progressed and knowledge of the COVID-19 pneumonia disease process has continued to advance, the safety and advantages of early tracheostomy have become more evident. The largest pooled data study to date, a systematic review and meta-analysis of 69 non-randomized studies including 4669 patients, assessed the benefits and timing of tracheostomy. This study showed a significant overall decrease in mortality in those patients who underwent tracheostomy in comparison to those who continued to be managed on mechanical ventilation via endotracheal tube. In addition, early tracheostomy and late tracheostomy (defined as before or after day fourteen of endotracheal intubation) were assessed.

It was determined that patients who underwent early tracheostomy had a shorter ICU length of stay but no significant difference in timing of weaning from mechanical ventilatory support or tracheostomy decannulation [7]. With regard to additional end-points after tracheostomy, a systematic review and meta-analysis by Benito et al. assessed the success of and time to tracheostomy decannulation in a pooled group of 3234 patients from eighteen studies and found that, while the overall post-tracheostomy mortality was 13.1%, nearly 35% of patients were successfully decannulated, with a mean decannulation time of 18.6 days [27].

More recent literature has shown that patients with COVID-19 once intubated have high rates of extubation failure with low use of prophylactic noninvasive ventilation (NIV). [28] Additional studies have shown that extubation to NIV can decrease the rates of reintubation but without increasing length of stay or mortality. [29]

Our study looked at tracheostomies’ outcomes for all COVID-19 patients at Mount Sinai Hospital based on the timing of the tracheostomy over a period of 1 year. This included the initial pandemic wave and then the second wave of SARS-CoV-2 in a major New York City hospital. During this time, our understanding of the disease evolved, as did the management. Our institution decided to follow our standard of care, with a goal of tracheostomy prior to day 12 for all our patients with prolonged mechanical ventilation [30]. This was carried out to both continue with the current practice at our institution as well as out of necessity. With the initial surge of patients, even while greatly expanding our ICU capacity, we would have very quickly run out of ICU beds without the ability to decompress the ICU [31]. The actual time to tracheotomy was influenced by several factors, including but not limited to high fio2 requirement on the vent and inability to tolerate the procedure, religious beliefs, and patient/family choice, etc. While we did our best to account for cofactors, it was not possible to account for the rapidly changing and evolving therapies and their influence on the overall outcome, as this is a retrospective analysis.

## 5. Conclusions

COVID-19 has been a worldwide pandemic, resulting in the loss of millions of people across the world. In March 2020, New York City became ground zero across the world in COVID-19 cases and deaths. At hospitals across New York City, the number of patients with severe ARDS and respiratory failure quickly overwhelmed the health system. At Mount Sinai Hospital, we surged from 104 ICU beds to 235 ICU beds [31]. With this being a new and unknown respiratory illness, there was a concern for the risk of performing tracheostomies prior to day 21 for the risk of infection to the physician team [30,32,33,34]. It was in this context that we, as an institute, decided to continue performing early tracheostomies when possible. We found that early tracheostomy, even in the patients with SARS-CoV-2 severe pneumonia, was safe, with no cases of known transmission to the operator. We also observed a reduction in the use of sedation, paralytics, and ICU days for these patients. As more effective therapies have been developed and a large amount of the population has been vaccinated, early tracheostomy has become a standard.

## Figures and Tables

**Table 1:** Demographics of patients admitted with COVID-19 to MSH.

Demographics of Patients Admitted with COVID-19 to MSH, Requiring ICU Stay, and Mechanical Ventilation			
Patients Admitted from 1 March 2020 to 1 March 2021			
Characteristic	All Patients	ICU Patients	Intubated Patients
	N = 3672 ^1^	N = 888 ^1^	N = 544 ^1^
Age	61(18)	62(16)	63(14)
Gender			
F	1649 (45%)	333 (38%)	200 (37%)
M	2022 (55%)	555 (62%)	344 (63%)
U	1 (<0.1%)	0 (0%)	0 (0%)
BMI	29(8)	30(8)	31(8)
Race			
White	1114 (30%)	239 (27%)	139 (26%)
Black	737 (20%)	156 (18%)	84 (15%)
Hispanic	849 (23%)	211 (24%)	140 (26%)
Asian	174 (4.7%)	47 (5.3%)	31 (5.7%)
Native American or Pacific Islander	21 (0.6%)	3 (0.3%)	3 (0.6%)
Other	777 (21%)	232 (26%)	147 (27%)
History of Diabetes	1434 (39%)	417 (47%)	274 (50%)
History of Hypertension	2228 (61%)	585 (66%)	365 (67%)
History of Chronic Lung Disease	678 (19%)	187 (21%)	106 (19%)
History of Chronic Liver Disease	320 (8.7%)	116 (13%)	77 (14%)
History of Renal Failure	746 (20%)	224 (25%)	137 (25%)
History of Heart Failure	602 (16%)	196 (22%)	122 (22%)
HIV/AIDS	77 (2.1%)	19 (2.1%)	14 (2.6%)
History of Alcohol or Substance Use Disorder	156 (4.3%)	49 (5.5%)	29 (5.3%)
BMI ≥ 30.0	1257 (39%)	367 (44%)	229 (44%)
Hospital Length of Stay in Days	7 (4, 14)	16 (9, 28)	20 (12, 34)
Died	618 (17%)	370 (42%)	313 (58%)
Discharged out of Hospital	3053 (83%)	518 (58%)	231 (42%)
Routine Discharge to Home	2496 (68%)	330 (37%)	97 (18%)
Not Routine Discharge to Home	557 (15%)	188 (21%)	134 (25%)

^1^ Mean (SD); n (%); median (IQR).

**Table 2:** Demographics of patients who underwent tracheostomy during the study period.

Demographics of Patients Who Underwent Tracheostomy during the Study Period			
Characteristic	Trach after Day 12, N = 103 ^1^	Trach on or before Day 12, N = 74 ^1^	*p*-Value ^2^
Age	61 (14)	63 (15)	0.12
Gender			0.5
Female	36 (35%)	22 (30%)	
Male	67 (65%)	52 (70%)	
Race			0.12
African American	14 (14%)	10 (14%)	
Asian Indian	4 (3.9%)	1 (1.4%)	
Bangladeshi	1 (1.0%)	1 (1.4%)	
Filipino	3 (2.9%)	0 (0%)	
Haitian	2 (1.9%)	0 (0%)	
Pakistani	1 (1.0%)	1 (1.4%)	
Trinidadian	0 (0%)	2 (2.7%)	
White	23 (22%)	24 (32%)	
Geography			0.6
Bolivian	1 (1.0%)	0 (0%)	
Dominican	4 (3.9%)	2 (2.7%)	
Ecuadorian	8 (7.8%)	2 (2.7%)	
Latin American	7 (6.8%)	4 (5.4%)	
Latina American	1 (1.0%)	1 (1.4%)	
Mexican	7 (6.8%)	2 (2.7%)	
Peruvian	0 (0%)	1 (1.4%)	
Puerto Rican	3 (2.9%)	4 (5.4%)	
South American Indian	1 (1.0%)	0 (0%)	
Spaniard	0 (0%)	1 (1.4%)	
Unknown/Other	74 (72%)	53 (72%)	
max_PaCO2	74 (20)	64 (17)	<0.001
max_D_DIMER	15.2 (15.4)	11.3 (8.0)	0.040
max_CREATININE	4.2 (4.0)	3.9 (3.2)	0.8
max_CRP	297 (108)	269 (107)	0.039
max_FERRITIN	3826 (4,666)	3452 (3,639)	0.8
min_PaO2	55 (17)	57 (11)	0.3
min_PFRatio_Streaming	79 (52)	82 (36)	0.2

^1^ Mean (SD); n (%). ^2^ Wilcoxon rank sum test; Pearson’s Chi-squared test; Fisher’s exact test.

**Table 3:** Outcomes for early vs. late tracheostomy.

	Early Tracheostomy (≤12 Days) n = 74	Late Tracheostomy (>12 Days) n = 103
Sedation pre-tracheostomy	9.64	8.13
Sedation post-tracheostomy	10.21	15.64
Total days on sedation	19.86	23.85
ICU LOS	23.66	67.91
Hospital LOS	33.2	88.8
	n = 48	n = 75
Days on paralytics	5.79	9.66
	n = 25	n = 43
Days on mechanical ventilator, intubation to date weaned	22.96	34.72
	n = 21	n = 36
Days on mechanical ventilator, adjusted for mortality	23.04	34.44

**Table 4:** Discharge disposition.

Group	Disposition							
	Acute Rehabn (%)	Deceased n (%)	Home n (%)	LTACH n (%)	SAR n (%)	SNF n (%)	Transfer to Another Hospital n (%)	Total
Early Trach	11 (6.21%)	38 (21.47%)	9 (5.08%)	12 (6.78%)	2 (1.13%)	1 (0.56%)	1 (0.56%)	74 (41.81%)
Late Trach	18 (10.17%)	40 (22.60%)	7 (3.95%)	24 (13.56%)	7 (3.95%)	3 (1.69%)	4 (2.26%)	103 (58.19%)
Total	29 (16.38%)	78 (44.07%)	16 (9.04%)	36 (20.34%)	9 (5.08%)	4 (2.26%)	5 (2.82%)	177 (100.00%)

## Data Availability

Dataset available on request from the authors.
